# The primary complement components contained in circulating immune complexes in oligoarticular and polyarticular juvenile idiopathic arthritis patient sera are C1q and C4: evidence of classical complement activation

**DOI:** 10.1186/1546-0096-10-S1-A120

**Published:** 2012-07-13

**Authors:** Brooke E Gilliam, Melinda R Reed, Anil K Chauhan, Amanda Dehlendorf, Sandra Crespo-Pagnussat, Terry L Moore

**Affiliations:** 1 St. Louis, MO, USA; 2 Saint Louis University, St. Louis, MO, USA

## Purpose

Circulating immune complexes (CICs) from juvenile idiopathic arthritis (JIA) sera have been shown to contain bound complement components; however, whether the classical or alternative pathway is the main pathway involved remains undetermined. To delineate the role of these pathways in the disease process, we measured activated complement products bound to CICs in sera from 100 JIA patients.

## Methods

Sera from 100 JIA patients were collected, including 68 polyarthritis (41 IgM RF-negative and 27 IgM RF-positive) and 32 oligoarthritis patients. Sera from 17 healthy children were also analyzed. C1q, C3, C3d, C4, and membrane attack complex (MAC) bound to CICs were measured by enzyme-linked immunosorbent assay (ELISA). IgA and IgM RF and IgG anti-cyclic citrullinated peptide (anti-CCP) antibodies were also measured by ELISA. C-reactive protein (CRP), erythrocyte sedimentation rate (ESR), and disease activity were obtained from patient records.

## Results

C1q and C4 bound to CICs had the highest percent of positivity in the JIA population (48% and 45%, respectively). C3 and C3d bound to CICs were positive in 36% and 25% of JIA patients, respectively. MAC bound to CICs was positive in 29% of patients. Levels of C1q, C4, C3 and MAC bound to CICs were all significantly elevated in JIA patients when compared to healthy controls (p<0.05). However, C3 levels remained normal in both JIA and healthy children. No significant differences were noted between JIA subtypes, with both polyarticular and oligoarticular subtypes favoring classical pathway activation. Strong correlations were noted between C1q and C4 bound to CICs (r=0.76), C1q and MAC bound to CICs (r=0.65), and C4 and MAC bound to CICs (r=0.66) (p<0.05). IgM RF correlated significantly with C4 bound to CICs (r=0.22, p=0.03). Of the 29 JIA patients positive for MAC bound to CICs, 27 (93%) were also positive for C1q bound to CICs, C4 bound to CICs, or both. Conversely, only 16/29 (55%) of JIA patients positive for MAC bound to CICs were positive for C3 bound to CICs, C3d bound to CICs, or both.

## Conclusion

These results indicate that the classical complement pathway is the primary pathway involved in the pathogenesis of JIA. While previous studies have implicated the classical pathway with smaller study populations, our study is one of the largest complement studies in JIA to indicate dominance of the classical pathway in JIA. Due to the amplification loop and higher levels of alternative pathway proteins in plasma, elevated levels of alternative pathway components are expected. MAC has received far less attention in JIA studies. Our study found 29% of JIA patients positive for MAC bound to CICs. These results largely the activation of the classical pathway by CICs in the sera from patients with JIA.

## Disclosure

Brooke E. Gilliam: None; Melinda R. Reed: None; Anil K. Chauhan: ProGen Biologics, LLC, 4; Amanda Dehlendorf: None; Sandra Crespo-Pagnussat: None; Terry L. Moore: None.

